# The Function of *Lgr5*^+^ Cells in the Gastric Antrum Does Not Require *Fzd7* or *Myc* In Vivo

**DOI:** 10.3390/biomedicines7030050

**Published:** 2019-07-08

**Authors:** Dustin Flanagan, Nick Barker, Matthias Ernst, Elizabeth Vincan, Toby Phesse

**Affiliations:** 1 University of Melbourne & Victorian Infectious Diseases Reference Laboratory, Doherty Institute of Infection and Immunity, Melbourne VIC 3000, Australia; 2 Institute of Medical Biology, Singapore 138648, Singapore; 3 MRC Centre for Regenerative Medicine, University of Edinburgh, Edinburgh EH8 9YL, UK; 4 Cancer and Inflammation Laboratory, Olivia Newton-John Cancer Research Institute and School of Cancer Medicine, La Trobe University, Heidelberg VIC 3084, Australia; 5 School of Pharmacy and Biomedical Sciences, Curtin University, Perth WA 6845, Australia; 6 European Cancer Stem Cell Research Institute, Cardiff University, Cardiff CF24 4HQ, UK

**Keywords:** Wnt, gastric stem cells, Frizzled-7, Fzd7, Myc, Lgr5

## Abstract

The extreme chemical and mechanical forces endured by the gastrointestinal tract drive a constant renewal of the epithelial lining. Stem cells of the intestine and stomach, marked by the cell surface receptor *Lgr5*, preserve the cellular status-quo of their respective tissues through receipt and integration of multiple cues from the surrounding niche. Wnt signalling is a critical niche component for gastrointestinal stem cells and we have previously shown that the Wnt receptor, *Frizzled-7* (*Fzd7*), is required for gastric homeostasis and the function of *Lgr5*^+^ intestinal stem cells. Additionally, we have previously shown a requirement for the Wnt target gene *Myc* in intestinal homeostasis, regeneration and tumourigenesis. However, it is unknown whether *Fzd7* or *Myc* have conserved functions in gastric *Lgr5*^+^ stem cells. Here we show that gastric *Lgr5*^+^ stem cells do not require *Fzd7* or *Myc* and are able to maintain epithelial homeostasis, highlighting key differences in the way Wnt regulates homeostasis and *Lgr5*^+^ stem cells in the stomach compared to the intestinal epithelium. Furthermore, deletion of *Myc* throughout the epithelium of the gastric antrum has no deleterious effects suggesting therapeutic targeting of Myc in gastric cancer patients will be well tolerated by the surrounding normal tissue.

## 1. Introduction

The epithelium of the gastrointestinal tract encounters substantial chemical and physical stresses. One of the mechanisms that has evolved to help cope with these harsh conditions is the continuous turnover of the epithelium, in which new cells are generated from populations of stem cells. These new cells then differentiate and migrate towards the lumen and are eventually sloughed off so that any damaged cells are not retained, and new healthy cells are being produced constantly to replace them. Lgr5 was first identified as a Wnt target gene and a marker of highly proliferative stem cells located at the base of the intestinal crypts that fuel the constant turnover of cells [1]. It has since been confirmed as a stem cell marker in several epithelial tissues including the stomach [2,3], hair follicle [4], ovary [5], mammary gland [6] and kidney [7].

Lgr5 is a receptor for secreted Wnt agonist R-spondins, which acts to modify the strength of Wnt signalling in cells, including those of the intestinal crypt [8]. Wnt signalling regulates several cell functions including proliferation, migration, apoptosis and differentiation, and is critical during embryonic development, and the homeostasis of several tissues including the intestine, stomach, liver and mammary gland [9]. Wnts are a family of 19 glycoproteins that are modified during the secretion process by an O-acyltransferase called Porcupine, which can then bind to Frizzled receptors of which there are 10 in mammals [10]. The fatty acid modification of Wnt ligands is critical for their bind to the U-shape of Frizzled dimers, which also associate with other co-receptors, including Lrp5/6 to form a signalosome with cytoplasmic Dishevelled. This signalosome then inhibits the action of a multimeric degradation complex which allows the co-transcription factor β-catenin to escape degradation and translocate into the nucleus and associate with TCF/Lef transcription factors to regulate target genes [11]. 

In the intestine, Wnt signalling is most active at the base of the crypts, which contain the *Lgr5+* stem cells and Paneth cells [1], with Wnt3a secreted from the Paneth cells [12], and Wnt2b from the underlying stroma [13]. We recently demonstrated that the deletion of *Fzd7* in *Lgr5^+^* intestinal epithelial cells was deleterious and triggered rapid repopulation with *Fzd7* proficient cells, indicating its requirement for this population of stem cells [14]. Similarly, the deletion of *Fzd7* throughout the antrum of the gastric epithelium also triggered repopulation, indicating that Fzd7 regulates a population of stem cells in the antrum [15]. Furthermore, *Fzd7* was highly expressed in gastric tumours, and transmitted Wnt signalling to upregulate the transcription factor *Myc*, to promote tumour initiation and growth [16]. This is consistent with the role of *Myc* in the intestinal epithelium in which it is required for homeostasis [17], regeneration [18] and tumorigenesis [19]. 

As *Lgr5* marks stem cells in several tissues it is important to understand how these populations of cells are regulated, and therefore here we asked if *Fzd7* and *Myc* regulate *Lgr5^+^* cells in the gastric antrum. 

## 2. Materials and Methods

### 2.1. Mice

The *Tff1Cre^ERT2^* [20], *Fzd7^fl/fl^* [14], *c-Myc^fl/fl^* [21], *Rosa26LacZ* [22] and *Lgr5Cre^ERT2^* [1] mice are previously described. Mice were interbred to generate compound mice with appropriate alleles on an inbred C57Bl/6 genetic background. Mice were co-housed using appropriate littermates as controls. All animal experiments were approved by the Animal Ethics Committee, Office for Research Ethics and Integrity, University of Melbourne (1513488, approved on 18th May 2015).

### 2.2. Treatments 

For short-term labelling, mice received a single daily intraperitoneal (IP) injection of 2 mg of tamoxifen. For long-term (>14 days) labelling, mice received single IP injections of 2 mg tamoxifen over three consecutive days.

### 2.3. Tissue Collection and Histological Analysis

Mouse stomachs were isolated, flushed with PBS, fixed overnight at 4 °C in 10% neutral buffered formalin (NBF) and processed for immunohistochemistry, as previously described [14,23,24]. List of antibodies used available upon request. 

### 2.4. β-Galactosidase (X-gal) Staining

Mouse stomachs were prepared and stained for X-gal as previously described [15].

### 2.5. Gland Isolation, Cell Dissociation and Organoid Culture 

Antral stomachs isolated from experimental mice were prepared for organoid culture as previously described [24]. Antral glands isolated for flow cytometry were prepared, as previously described [3]. 

### 2.6. Genomic Recombination PCR

Conventional PCR to detect the *Fzd7* and *c-Myc* mutant alleles following recombination in genomic DNA extracted from compound transgenic mice was performed, as previously described [14,19]. 

### 2.7. RNA Extraction and Analysis 

Whole antral glands and FACS-isolated single antral cells were homogenized in TRizol and total RNA purified, DNAse treated, quantified and subjected to quantitative reverse transcriptase PCR (qRT-PCR). qRT-PCR and calculating gene expression levels relative to the house-keeping gene 18S (2^−∆∆*C*t^) were performed as previously described [25]. 

### 2.8. MTT Assay

Following treatment, gastric organoids were mechanically dissociated, washed with ADF, resuspended in fresh Matrigel and seeded in a flat bottom 96 well tissue culture plate for enumeration using the MTT assay performed exactly as we previously described [14,15].

### 2.9. Statistical Analysis

Data are expressed as mean ± SEM, where the mean represents the number of mice (≥3 per genotype) or number of independent experiments (≥3). Statistical tests used were two-way ANOVA with Prism7 (GraphPad software) where *p* values of ≤0.05 were considered significant. 

## 3. Results

**Figure 1:**
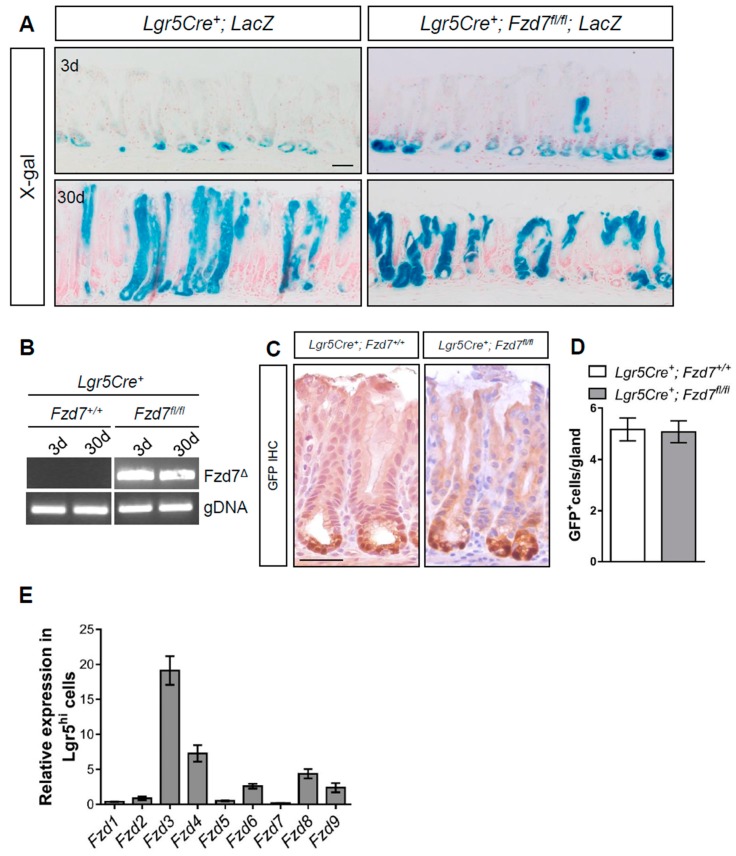
Conditional deletion of *Fzd7* from Lgr5^+^ gastric stem cells does not perturb homeostasis. (**A**) X-gal stained tissue sections from *Lgr5Cre^+^*; *LacZ* and *Lgr5Cre^+^*; *Fzd7^fl/fl^*; *LacZ* mice 3 and 30 days following tamoxifen. Scale bars = 100 μm. (**B**) Conventional PCR for recombined *Fzd7* (Fzd7^Δ^) alleles in mice described in (**A**). (**C**) Representative IHC staining for GFP, which permits detection of *Lgr5^GFP+^* stem cells, on sections from mice described in A at 30 days post tamoxifen. Scale bars = 100 µm. (**D**) Quantification of GFP^+^ cells in sections from mice described in A (mean ± SEM, *n* = 3 mice, at least 30 glands scored/mouse, Unpaired student *t*-test). (**E**) qPCR for Fzd receptors in FACS-isolated GFP+ (Lgr5^hi^) cells. Expression was normalized to Lgr5^lo^ cells.

To determine the requirement for *Fzd7* in antral *Lgr5^+^* cells we deleted *Fzd7* specifically in *Lgr5^+^* cells using *Lgr5Cre^ERT2^*; *Fzd7^fl/fl^*; *LacZ^LSL^* mice and performed lineage tracing via X-gal staining. At 3 days post tamoxifen induction recombined cells of *Fzd7* proficient *Lgr5Cre^ERT2^*; *Fzd7^+/+^*; *LacZ^LSL^* mice can be seen at the base of the antral crypts where the *Lgr5^+^* cells are located, and 30 days after tamoxifen many of the crypts are composed entirely of lineage traced cells indicating the variegated Lgr5 locus gives rise to entire gastric units in the antrum as previously reported [2] (Figure 1A). Surprisingly, lineage tracing also proceeded in the *Fzd7* deficient *Lgr5Cre^ERT2^*; *Fzd7^fl/fl^*; *LacZ^LSL^* mice indicating that *Fzd7* loss is not deleterious to *Lgr5^+^* cells in the gastric antrum, and these stem cells can function without *Fzd7*. To confirm robust deletion of *Fzd7* we performed PCR for the recombined product which gave a very strong band 3 and 30 days after tamoxifen induction in *Lgr5Cre^ERT2^*; *Fzd7^fl/fl^*; *LacZ^LSL^* mice, whilst in *Lgr5Cre^ERT2^*; *Fzd7^+/+^*; *LacZ^LSL^* mice the recombined product was undetectable as expected (Figure 1B). The *Lgr5Cre^ERT2^* locus also has an EGFP cassette and therefore we performed immunohistochemistry (IHC) for GFP which demonstrated no difference in the number of *Lgr5^+^* cells in the gastric antrum after *Fzd7* deletion (Figure 1C,D). Together these data demonstrate that, in contrast to the intestinal epithelium, *Fzd7* is not required for the activity of *Lgr5^+^* stem cells in the gastric antral epithelium. Indeed, RT-qPCR for *Fzd* genes in cells FACS sorted for high GFP expression from the antrum epithelium of *Lgr5Cre^EGFP-ERT2^* mice revealed that Fzd7 expression was undetectable (Figure 1E), thus supporting our in vivo observations that *Lgr5^+^* cells in the gastric antrum are regulated differently from those in the intestinal epithelium, and do not require *Fzd7*.

**Figure 2:**
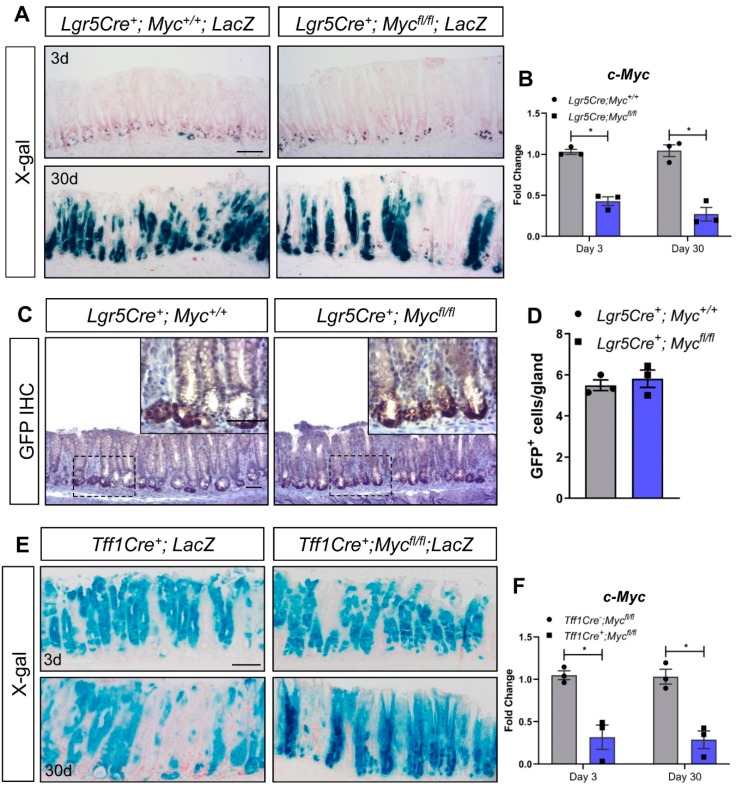

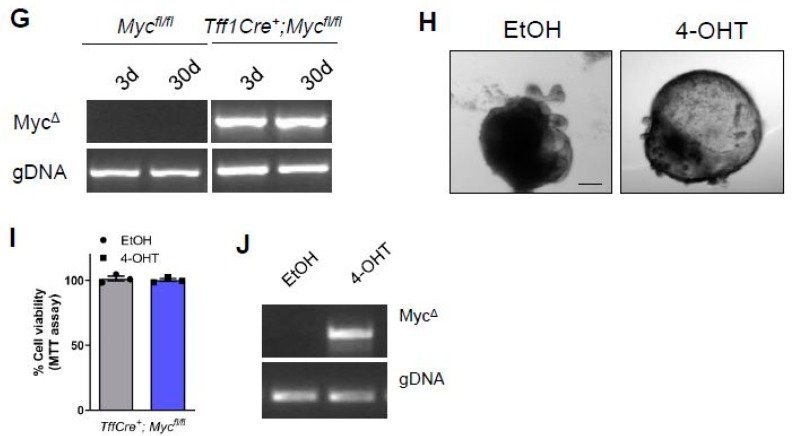
Conditional deletion of *Myc* from the gastric epithelium does not trigger epithelial repopulation. (**A**) X-gal stained tissue sections from *Lgr5Cre^+^*; *LacZ* and *Lgr5Cre^+^*; *Myc^fl/fl^*; *LacZ* mice 3 and 30 days following tamoxifen. Scale bars = 100 μm. (**B**) qRT-PCR for c-*Myc* on gastric epithelial cells isolated from *Lgr5Cre^+^*; *Myc^+/+^* and *Lgr5Cre^+^*; *Myc^fl/fl^* mice following tamoxifen at 3 and 30 days post tamoxifen as indicated (* *p* < 0.05, mean ± SEM, *n* = 3 mice, Two-way ANOVA). (**C**) Representative IHC for GFP, which permits detection of *Lgr5^GFP+^* stem cells, on sections from mice described in A. Scale bars = 100 µm. (**D**) Quantification of GFP^+^ cells in sections from mice described in A (mean ±SEM, *n* = 3 mice, at least 30 glands scored/mouse, unpaired student *t*-test). (**E**) X-gal stained tissue sections from *Tff1Cre^+^*; *LacZ* and *Tff1Cre^+^*; *Myc^fl/fl^*; *LacZ* mice 3 and 30 days following tamoxifen. Scale bars = 100 μm. (**F**) qRT-PCR for c-*Myc* on gastric epithelial cells isolated from *Tff1Cre^-^*; *Myc^fl/fl^* and *Tff1Cre^+^*; *Myc^fl/fl^* mice following tamoxifen (* *p* < 0.05, mean ± SEM, *n* = 3 mice, two-way ANOVA). (**G**) Conventional PCR for recombined *Myc* (Myc^Δ^) alleles in mice described in (**E**). (**H**) Representative DIC images of gastric organoids derived from *Tff1Cre^+^*; *Myc^fl/fl^* mice 5 days after treatment with vehicle (EtOH) or tamoxifen (4-OHT). Scale bars = 100 µm. (**I**) MTT viability assay performed on organoid cultures described in (**G**) (mean ±SEM, *n* = 3 biological replicates, unpaired student *t*-test). Individual experiments were repeated twice. (**J**) Conventional PCR for recombined *Myc* (Myc^Δ^) alleles on organoid cultures described in (**H**).

We have previously shown that deletion of *Myc* in the intestinal epithelium phenocopies deletion of *Fzd7* and results in rapid repopulation with *Myc* proficient cells [17]. Furthermore, we recently demonstrated that Wnt regulates *Myc* expression, via Fzd7, to control tumour initiation and growth in the stomach [16], regeneration in the intestine [14] and homeostasis in the stomach [15]. To investigate if *Lgr5^+^* cells in the antrum require Myc, we deleted *Myc* in *Lgr5^+^* cells in vivo. Surprisingly, and similar to deletion of *Fzd7*, fully lineage traced gastric units were observed in the antrum of *Lgr5Cre^ERT2^; Myc^fl/fl^; LacZ^LSL^* mice 30 days after tamoxifen induction (Figure 2A), with no difference in the number of *Lgr5^+^* cells between *Lgr5Cre^ERT2^*; *Myc^fl/fl^* and *Lgr5Cre^ERT2^*; *Myc^+/+^* mice (Figure 2B,C). These data demonstrate that *Lgr5^+^* antral cells do not require *Myc* for their stem cell activity. However, there are several stem cell populations identified in the antrum based on expression of distinct maker genes including *Lrig1* [26], *Sox2* [27] and *CCK2R* [28] and therefore *Myc* could be required for activity of one of these populations of stem cells which would have been missed in the analysis of our *Lgr5Cre* mice. 

To investigate if Myc is required generically in the antral epithelium we deleted *Myc* throughout the antral epithelium using *Tff1Cre^ERT2^* mice [15]. X-Gal staining revealed robust recombination throughout the antral epithelium of *Tff1Cre*; *LacZ^SLS^* mice 3 days after tamoxifen induction (Figure 2D). Recombination was still observed at 30 days post induction (Figure 2D), demonstrating that recombination has occurred in at least one population of antral stem cells as previously published [15]. Remarkably, deletion of *Myc* throughout the antral epithelium of *Tff1Cre*; *Myc^fl/fl^*; *LacZ^SLS^* mice did not perturb stem cell activity and lineage tracing was able to proceed as per *Tff1Cre*; *Myc^+/+^*; *LacZ^SLS^* mice (Figure 2D) despite confirming robust deletion of *Myc* in the antral epithelium via RT-qPCR (Figure 2E), and retention of a strong recombined band via PCR 3 days and 30 days after tamoxifen induction of *Tff1Cre*; *Myc^fl/fl^*; *LacZ^SLS^* mice (Figure 2F). To help confirm this observation that the antral epithelium can function in the absence of *Myc*, we cultured gastric organoids from the antrum of *Tff1Cre*; *Myc^fl/fl^* mice, and deleted *Myc* via treatment with 4-OHT (we have previously demonstrated that 4-OHT does not adversely affect gastric organoids [15]. *Myc* deleted organoids continued to thrive (Figure 2G) and MTT assays showed no difference in viability compared to vehicle-treated, *Myc* proficient organoids (Figure 2H), despite confirming robust recombination of the *Myc* flox allele (Figure 2I). Together these data demonstrate that the epithelium of the gastric antrum does not require *Myc* in vivo, or in cultured organoids.

## 4. Discussion

*Fzd7* is required for intestinal stem cell activity during homeostasis, regeneration and survival of cultured organoids [29]. Here we show for the first time that deletion of *Fzd7* does not inhibit the capacity of *Lgr5+* cells to lineage trace full gastric units in the antral epithelium illustrating a substantial difference for *Fzd7* in regulating *Lgr5+* stem cells in the intestine compared to the antrum. We also show that the number of *Lgr5+* cells is maintained in the *Fzd7* deficient antrum at similar levels to that of *Fzd7* proficient mice, demonstrating that lineage tracing is not due to a small population of *Lgr5+* cells that were resistant to *Fzd7* deletion, but rather that *Fzd7* deletion has not affected *Lgr5+* survival and activity. We also observe that other *Fzd* genes are expressed in *Lgr5^HI^* cells in the antrum, with *Fzd3* and *Fzd4* the highest, suggesting one of these may be transmitting Wnt signalling in antral cells. Interestingly, the expression pattern for *Fzd3* seems to be consistent with that of *Lgr5* in the gastric antrum whilst *Fzd4* is expressed broadly throughout the gastric units [30]. However, *Fzd3* is significantly upregulated when *Fzd7* is deleted in gastric antrum organoids, but this increased expression is unable to compensate for the loss of *Fzd7* in vitro and *Fzd7*-deficient organoids undergo apoptosis [15]. Thus, the exact Fzd receptors required for antral *Lgr5+* cell activity will require additional functional studies in the future including analysis of Wnt pathway activity. We have previously shown that the deletion of Fzd7 throughout the epithelium of the gastric antrum is deleterious and triggers rapid repopulation [15]. This demonstrates that Fzd7 is required for at least one population of stem cells in the gastric antrum, but this population has yet to be identified, and our data here illustrate it is not *Lgr5+* antral stem cells. A possible candidate for this population is *Axin2+/Lgr5-* cells which are located in a similar location as *Fzd7* expressing cells in the lower half of the antral glands [30]. Furthermore, the *Axin2+/Lgr5-* population in the antrum expands in response to *Helicobacter pylori* infection via upregulation of *Rspo3* in the underlying myofibroblasts [30], whilst inhibition of Fzd7 can suppress *H. pylori*-induced Wnt signalling and proliferation [31]. These data suggest Fzd7 may be regulating *Axin2+/Lgr5-* cells in the antrum, although this is yet to be confirmed.

We have previously shown that *Myc* is required for all the tumourigenic phenotypes following truncation of *Apc* in the intestine [19], and also for homeostasis [17], regeneration [18] and apoptosis [32] in this tissue. Furthermore, we recently demonstrated that tumour initiation and growth in the gastric antrum requires *Fzd7* dependant upregulation of *Myc* [16]. *Myc* is upregulated in many cancers and thus represents a potential target for therapy which has been the subject of intense research for several years. However, *Myc* is not required in all the adult tissues it is expressed in, for example, both liver zonation and *Apc* loss induced hepatomegaly are regulated by Wnt signalling via *Myc* independent mechanisms [33,34]. Given its differential requirement between different tissues it is important to gain a full insight into how the loss of *Myc* affects separate tissues. To our surprise, *Myc* deletion did not inhibit stem cell activity in *Lgr5^+^* stem cells of the gastric antrum or when genetically deleted throughout the entire epithelium of the gastric antrum. Furthermore, organoids cultured from the antrum were able to thrive and showed no difference in viability when *Myc* was deleted compared to *Myc* proficient organoids. These data demonstrate that *Myc* is not only dispensable for antral *Lgr5^+^* stem cells, but the entire antral epithelium. 

These data identify a distinct difference in the way Wnt regulates homeostasis and stem cells in the gastric antrum compared to the intestinal epithelium. As *Myc* is required for gastric tumour growth, it also informs future clinical trials with putative Myc inhibitors that the gastric epithelium can tolerate the loss of *Myc*. Future investigations into the differences between the intestinal and gastric epithelium could help uncover why the intestine is more sensitive to deregulated Wnt, and explore if Wnt inhibitors elicit a different response from these two tissues thus identifying new oncogenes/tumour suppressors in both cancer types.

Intestinal-type gastric tumours undergo metaplasia to become more intestinal-like, with upregulation of intestinal specific genes and morphological features including tubular structures. This could explain why gastric adenomas are sensitive to deletion of *Myc*, whilst the normal gastric epithelium is not, as the adenoma has acquired features of the intestinal epithelium which is sensitive to *Myc* loss. However, the exact molecular mechanism behind this observation has yet to be identified and requires further comparative experiments to delineate the role of Wnt/Fzd/Myc in both the normal and transformed tissue of the stomach and intestine.
